# Heat-Killed *Bifidobacterium longum* BBMN68 in Pasteurized Yogurt Alleviates Mugwort Pollen-Induced Allergic Airway Responses through Gut Microbiota Modulation in a Murine Model

**DOI:** 10.3390/foods12102049

**Published:** 2023-05-19

**Authors:** Xiaokang Niu, Xindi Yin, Xiuying Wu, Qi Zhang, Yunyun Jiang, Jingjing He, Yuyang Zhao, Chao Zhang, Yimei Ren, Mengxuan Lai, Yue Sang, Ran Wang

**Affiliations:** 1 Key Laboratory of Functional Dairy, Department of Nutrition and Health, China Agricultural University, Beijing 100190, China; 2 Inner Mongolia Mengniu Dairy (Group) Co., Ltd., Hohhot 011500, China; 3 College of Food Science & Nutritional Engineering, China Agricultural University, Beijing 100083, China; 4 Hebei Engineering Research Center of Animal Product, Sanhe 065200, China

**Keywords:** airway allergic inflammation, *Bifidobacterium longum* BBMN68, heat-killed bacteria, gut microbiota, gut-lung axis, pollen allergy, postbiotics

## Abstract

Many probiotic bacteria have been proven to prevent allergic airway responses through immunomodulation. This study was conducted to evaluate the effects of heat-killed *Bifidobacterium longum* BBMN68 (BBMN68) in pasteurized yogurt on the alleviation of mugwort pollen (MP)-induced allergic inflammation. BALB/c mice aged 5–6 weeks were randomly assigned and fed pasteurized yogurt containing heat-killed BBMN68 for 27 days, followed by allergic sensitization and challenge with MP extract. The allergic mice that received pasteurized yogurt containing heat-killed BBMN68 had improved immune status, including a lower serum IgE level, decreased serum interleukin (IL)-4, IL-5, and IL-13 concentrations, and alleviated airway inflammation manifested by increased macrophage and decreased eosinophil and neutrophil counts in BALF, as well as airway remodeling and suppressed peribronchial cellular infiltration. Moreover, oral administration of pasteurized yogurt containing heat-killed BBMN68 significantly modulated gut microbiota composition by influencing the proportion of beneficial genera associated with inflammation and immunity, such as *Lactobacillus*, *Candidatus_Saccharimonas*, *Odoribacter*, and *Parabacteroides*, which also negatively correlated with serum IgE and Th2 cytokine levels. These results demonstrated that pasteurized yogurt containing heat-killed BBMN68 had mitigative effects on allergic airway inflammation, likely through maintaining the systemic Th1/Th2 immune balance by altering the structure and function of the gut microbiota.

## 1. Introduction

Allergic airway disease is characterized by airway structure and function deterioration induced by T helper 2 (Th2) responses derived from adaptive immune normality due to harmless environmental stimuli such as pollen [1]. Mugwort pollen (MP) is one of the most common aeroallergens [2]. The dominant allergenic protein Art v 1 of mugwort pollen triggers pro-inflammatory Th2-associated cytokines that result in Th1/Th2 imbalance and the following allergic symptoms such as IgE secretion, mucus hypersecretion, eosinophilic inflammation, and airway hyperresponsiveness (AHR), leading to life quality impairment and extra economic costs [3]. The development of allergic airway disease is usually accompanied by gut microbiome shifts [4]. Therefore, preventive and treatment approaches through modulation of gut microbiota have attracted increasing attention.

Probiotics are living microorganisms that benefit the hosts gut microbiome [5]. They are able to enhance the gut microbiome’s resilience as well as local and systemic immune function [4,6,7,8]. *Lactobacillus* and *Bifidobacterium* are the most well-studied probiotics with strain-specific properties of immunomodulation and immunostimulation. Research on supplementation of live *Lactobacillus rhamnosus* GG (LGG) has verified its potential as a promising strategy for allergy prevention by decreasing serum IgE levels, lung eosinophil counts, lung Th2 cytokines, AHR, lung matrix metalloproteinase 9 expression, inflammatory cell infiltration, and the concentration of exhaled nitric oxide [9,10,11]. In recent years, postbiotics, defined as “preparation of inanimate microorganisms and/or their components that confers a health benefit on the host”, have been employed to mimic the effects of probiotics and overcome the challenges in their application, such as the instability and safety issues of live cells [12]. Both live and heat-killed LGG have shown anti-inflammatory effects in different models [13,14]. Administration of heat-killed *Clostridium butyricum* CGMCC0313-1 through aerosol inhalation was shown to adjust the Th1/Th2 balance and suppress the NF-κB/NLRP3 inflammatory pathway in the lungs of allergic mice [15]. Heat-killed *Lactobacillus plantarum* and *Lactobacillus curvatus* inhibited AHR by reducing cytokine levels and inducing regulatory factor Foxp3 expression [16]. The immunological effects of heat-killed probiotic bacteria suggest their important roles in relieving pollen allergies.

*Bifidobacterium longum* BBMN68 (BBMN68) is a probiotic strain isolated from healthy Chinese centenarians [17]. It has previously been shown to boost innate and acquired immune functions, including enhancement of macrophages and natural killer cell activities, induction of CD11c+ CD103+ dendritic cells (DC) and semi-mature DCs, and proliferation of splenic lymphocytes [18,19,20]. Moreover, the immunomodulatory benefits of BBMN68 are primarily derived from the cell wall fraction [19]. Therefore, it is proposed that heat-killed BBMN68 has potential for pollen allergy alleviation by modulating immune function. In this study, we investigated the effects of oral administration of heat-killed BBMN68 in pasteurized yogurt on the mitigation of allergic symptoms through interventions on the immune system and gut microbiota in MP-induced mice.

## 2. Materials and Methods

### 2.1. Preparation of the Pasteurized Yogurt Containing Heat-Killed BBMN68

*B. longum* BBMN68 (CGMCC 2265) was obtained from the China General Microbiological Culture Collection (CGMCC, Beijing, China). As a comparison, *Lactobacillus rhamnosus* GG (LGG, ATCC 53103) was purchased from the American Tissue Culture Collection (ATCC, Virginia, MA, USA). Both strains were cultured in MRS (De Man, Rogosa, and Sharpe) broth (Land Bridge Technology Co., Ltd., Beijing, China) at 37 °C for 24 h. Bacterial cells were centrifuged and washed before heat-inactivation in a water bath at 85 °C for 30 min.

All test yogurts were fermented with *Lactobacillus bulgaricus* and *Streptococcus thermophilus* based on a common procedure for conventional production and then pasteurized at 75 °C for 1 min. The heat-killed BBMN68 or LGG were additionally supplemented into the pasteurized yogurt at a concentration of 10^8^ cells/mL. It contains 78 kcal, 5.3 g of carbohydrate, 3.5 g of fat, and 3.0 g of protein per 100 g of yogurt.

### 2.2. Animals and Experimental Design

A total of 40 male BALB/c mice (Vital River Laboratories, Beijing, China) at an age of 5–6 weeks were randomly assigned to five groups, with 8 mice in each group. Mice were housed in a specific-pathogen-free (SPF) facility with free access to standard pelleted laboratory chow and water under standard conditions (12 h light/dark cycle, 20 ± 2 °C, 45–50% humidity). The animal management and experimental procedures were approved by the Animal Care and Use Ethics Committee (PONY-2021-FL-72).

**Figure 1:**
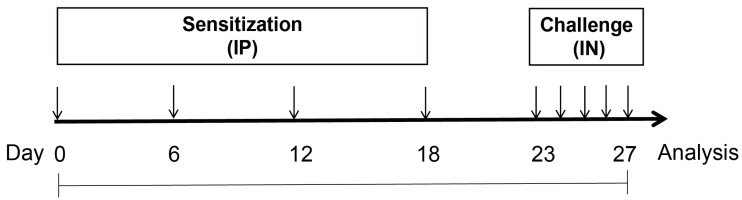
Procedures for establishing the model of mugwort pollen (MP)-induced allergic sensitization in mice. Mice received intraperitoneal (IP) injections of 0.2 mg MP for sensitization. The challenge was performed intranasally (IN) with 0.32 mg MP. Mice in the control group received saline for sensitization and challenge.

The procedures for establishing the model of mugwort pollen (MP)-induced allergic sensitization in mice are shown in Figure 1, as previously described with slight modifications [2]. Briefly, mice received four intraperitoneal injections of 0.1 mL MP (Aileji Biotechnology Co., Ltd., Hangzhou, China) at 2 mg/mL in the presence of 5% aluminum hydroxide (Thermo Fisher Scientific, Waltham, MA, USA) on days 0, 6, 12, and 18 for sensitization. Then mice were challenged intranasally with 40 μL MP at 8 mg/mL on days 23, 24, 25, 26, and 27. Mice in the control group received an equal amount of saline for sensitization and provocation and had daily administration of 0.2 mL of phosphate buffered saline (PBS, Solarbio, Beijing, China) during the whole experimental period (control group). Mice in the other four MP-induced allergic groups received 0.2 mL of PBS (MP group), pasteurized plain yogurts (Y + MP group), pasteurized yogurt containing heat-killed BBMN68 (BBMN68 + MP group), or pasteurized yogurt containing heat-killed LGG (LGG + MP group) intragastrically.

On day 28, mice were fasted for 12 h and anesthetized through an intraperitoneal injection of 10% chloral hydrate. Bronchoalveolar lavage fluid (BALF) was acquired by lavaging the lungs three times with 0.8 mL of sterile PBS. Blood and lung tissue samples were collected. Fecal pellets were freshly collected and stored on ice, then immediately transported to the laboratory for 16S rRNA gene sequencing.

### 2.3. Determination of Serum Immunoglobulins and Cytokines

Total serum IgE, IgG, Th2-associated cytokines IL-4, IL-5, IL-13, Th1-associated cytokine IFN-γ, and Th17-associated cytokine IL-17 were determined by enzyme-linked immunosorbent assay (ELISA) using commercial kits (Neobioscience Technology, Shenzhen, China; Multisciences Biotech, Hangzhou, China). All experimental procedures were conducted following the manufacturer’s instructions.

### 2.4. The Differential Cell Counts in BALF

After centrifugation, the precipitated BALF was resuspended with PBS. The cell counts of macrophages, eosinophils, neutrophils, and lymphocytes in BALF were observed on a glass slide stained with hematoxylin and Congo Red (Solarbio, Beijing, China) using a Leica DM 2500 microscope (Leica, Wetzlar, Germany). The percentage of differential cells was calculated for 200 cells.

### 2.5. Lung Histology and Hematoxylin-Eosin Staining

The left lung was fixed with 4% paraformaldehyde for 24 h and embedded in paraffin. The tissue sections were cut into 5 μm thick using a microtome and stained with hematoxylin and eosin (HE). The inflammatory cell infiltration was assessed at 20× magnification under a light microscope (Leica, Wetzlar, Germany). Then the semi-quantitative analysis of inflammation severity was scored based on the area of infiltration sites in the lung tissue sections: score 0 (no inflammation); score 1 (≤1/3 tissue involvement); score 2 (1/2 tissue involvement); and score 3 (≥2/3 tissue involvement) [21].

### 2.6. Fecal Microbiota Analysis

The genomic DNA of fecal samples was extracted using the MOBIO Power Fecal DNA Isolation Kit (Qiagen, Stockach, Germany) according to the instructions. The amplification of the V3–V4 region of the 16S rRNA gene was performed with the PCR bar-coded primer pair 338F (5′-ACTCCTACGGGAGGCAGCAG-3′) and 806R (5′-GGACTACHVGGGTWTCTAAT-3′). PCR products were purified with the AxyPrep DNA Gel Extraction Kit (Axygen Biosciences, Union City, CA, USA) after agarose gels electrophoresis. The purified amplicons were subsequently pooled in equimolar amounts and paired-end sequenced on an Illumina MiSeq PE300 platform (Illumina, San Diego, CA, USA).

The sequencing raw data were demultiplexed, quality-filtered by fastp (version 0.20.0), and merged by FLASH (version 1.2.7). Operational taxonomic units (OTUs) with 97% similarity were clustered using UPARSE (version 7.1), and taxonomies were analyzed using the RDP Classifier (version 2.2). The rarefaction analysis, including the Shannon, Ace, and Chao indices, was performed to demonstrate the α-diversity. Principal coordinate analysis (PCoA) was performed with the Quantitative Insights into Microbial Ecology (QIIME) pipeline (version 1.9.0). The correlation between gut microbiota and allergic sensitization parameters in serum was revealed using Pearson correlation. A differential abundance of taxa was identified using the linear discriminant analysis effect size (LEfSe).

### 2.7. Statistical Analysis

Statistical analysis was performed using a one-way ANOVA followed by Tukey’s multiple comparison test to compare groups on the SPSS software (version 25.0, IBM Corp., Armonk, NY, USA). Data were plotted with the GraphPad Prism 8.0.1 software (GraphPad Software Inc., Boston, MA, USA) and expressed as mean ± standard error of the mean (SEM). A *p*-value < 0.05 was considered significant.

## 3. Results

### 3.1. Protective Effects of Heat-Killed BBMN68 in Pasteurized Yogurt on Allergic Airway Responses

**Figure 2:**
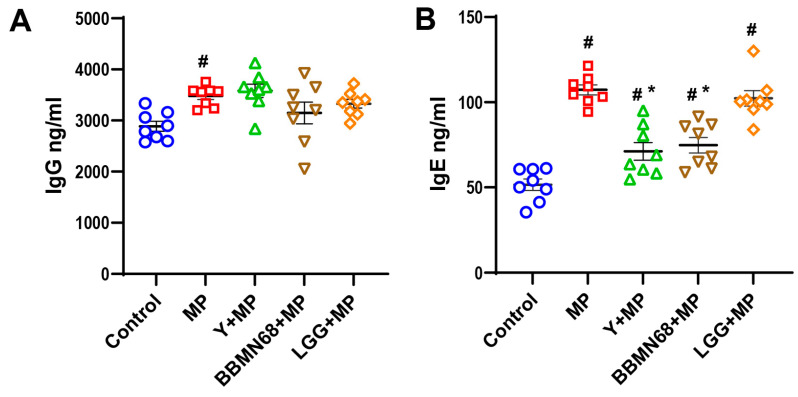
Serum IgG (**A**) and IgE (**B**) antibody levels in mice treated with pasteurized plain and heat-killed BBMN68 or LGG yogurt exposed to mugwort pollen (MP). Data are expressed as mean ± SEM, different color graphics in each group represent duplicates. #: *p* < 0.05 compared to the control group; *: *p* < 0.05 compared to the MP group; *n* = 8.

The protective effects of pasteurized yogurt containing heat-killed bacteria were tested using a MP-induced allergic sensitization mouse model, as depicted in Figure 1. After sensitization and challenge, the serum antibody IgE and IgG levels were measured (Figure 2). Compared with the control group, the total IgE and IgG in the serum of MP-exposed mice were increased (*p* < 0.05), indicating that MP induced the shift of the systemic immune balance toward Th2 immune responses. The allergic mice that received pasteurized plain and BBMN68 yogurt had lower (*p* < 0.05) serum IgE levels compared to the MP group, whereas the effect was not observed in the LGG + MP group.

**Figure 3:**
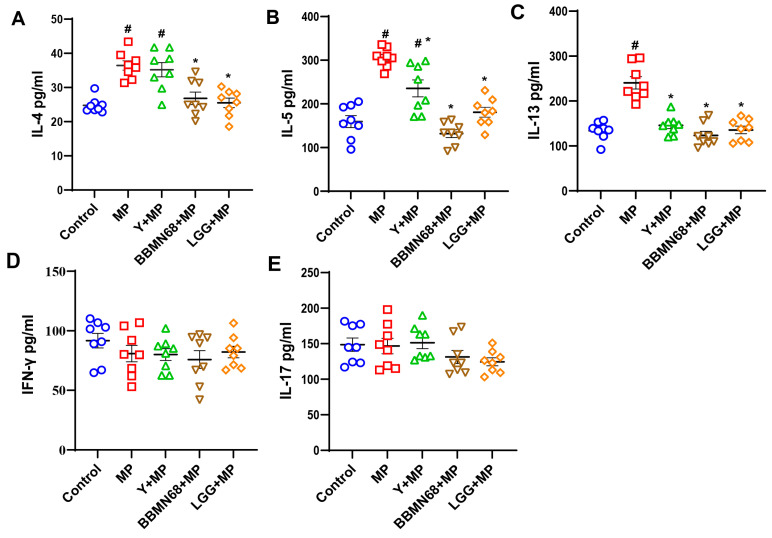
Serum cytokines of mice treated with pasteurized plain and heat-killed BBMN68 or LGG yogurt exposed to mugwort pollen (MP). (**A**) IL-4, (**B**) IL-5, (**C**) IL-13, (**D**) IFN-γ, (**E**) IL-17. Data are expressed as mean ± SEM, different color graphics in each group represent duplicates. #: *p* < 0.05 compared to the control group; *: *p* < 0.05 compared to the MP group; *n* = 8.

The Th2-associated cytokines IL-4, IL-5, IL-13, Th1-associated cytokine IFN-γ, and Th17-associated cytokine IL-17 in serum were also determined (Figure 3A–E). The IL-4, IL-5, and IL-13 levels in the serum of MP-exposed mice were significantly higher than those in the control group (*p* < 0.05). In comparison with the MP group treated with no yogurt, oral administration of heat-killed BBMN68 or LGG pasteurized yogurt reduced (*p* < 0.05) serum IL-4 and IL-5 levels. In addition, as shown in Figure 3C, pasteurized yogurt with and without BBMN68 or LGG all played a significant role in decreasing (*p* < 0.05) the IL-13 level in allergic mice. No significant difference was identified in serum IFN-γ and IL-17 levels.

**Figure 4:**
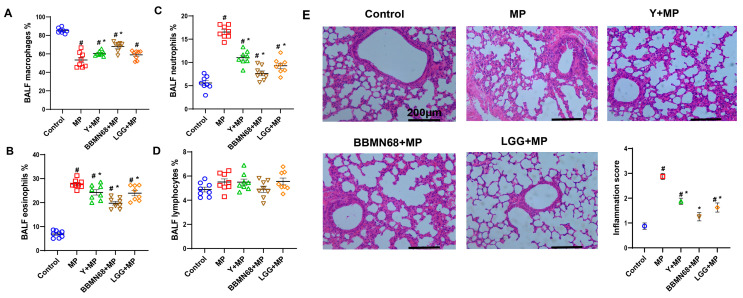
The BALF differential cell counts (%) of mice treated with pasteurized plain and heat-killed BBMN68 or LGG yogurt exposed to mugwort pollen (MP). (**A**) macrophages, (**B**) eosinophils, (**C**) neutrophils, (**D**) lymphocytes per 200 cells; (**E**) representative images of left lung stained with H and E, scale bars are 200μm; semi-quantitative scoring of inflammation in lung tissues. Data are expressed as mean ± SEM, different color graphics in each group represent duplicates. #: *p* < 0.05 compared to the control group; *: *p* < 0.05 compared to the MP group; *n* = 8.

The effects of heat-killed BBMN68 yogurt on allergic airway inflammation were evaluated by BALF differential cell counts and lung histology (Figure 4A–E). The MP-induced allergic mice had lower (*p* < 0.05) macrophage and higher (*p* < 0.05) eosinophil and neutrophil counts than mice in the control group. The significantly increased (*p* < 0.05) macrophage counts were observed in allergic mice that received pasteurized plain and BBMN68 yogurt compared to mice in the MP group; among them, the macrophage count was highest in the BBMN68 + MP group. Both neutrophil and eosinophil counts decreased (*p* < 0.05) in allergic mice that received pasteurized plain, BBMN68, or LGG yogurt. Moreover, the HE staining of left lung tissue sections indicated different pasteurized yogurt treatments in allergic mice promoted airway remodeling and suppressed peribronchial cellular infiltration, manifested by a lower inflammation score (*p* < 0.05), especially in the mice gavaged with heat-killed BBMN68 yogurt (Figure 4E).

### 3.2. Intervention of Heat-Killed BBMN68 in Pasteurized Yogurt on Gut Microbiota Composition Systemically Correlates with Allergic Airway Response

**Figure 5:**
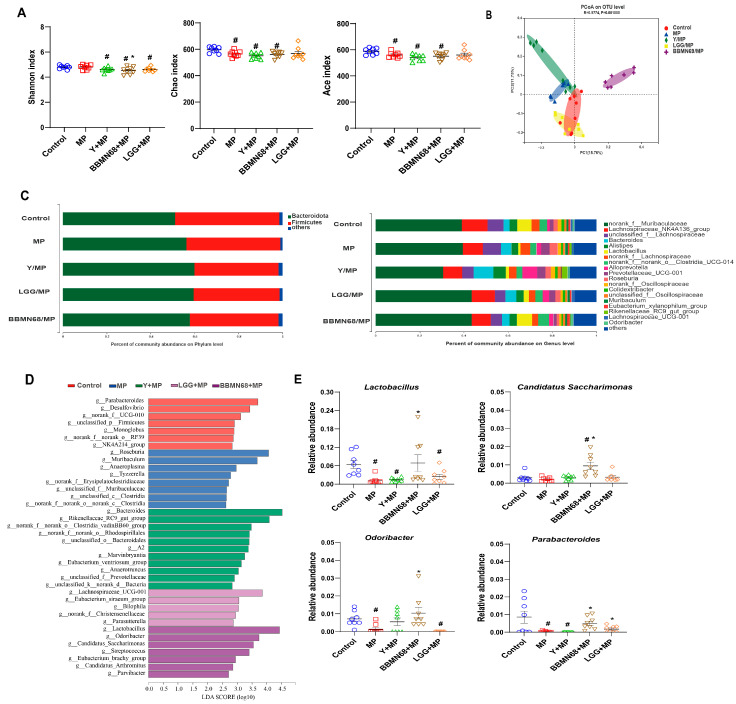
Effects of oral administration of pasteurized plain and heat-killed BBMN68 or LGG fermented yogurt on the fecal microbiota of allergic mice exposed to mugwort pollen (MP). (**A**) Alpha-diversity was calculated with the Shannon index, Chao index, and Ace index. (**B**) PCoA plot based on the unweighted-unifrac distances of the OTUs. (**C**) Percentage of community abundance on phylum and genus levels. (**D**) The differentially abundant taxa identified by LEfSe with LDA values (LDA score > 2.5). (**E**) Comparison of relative abundances of significantly different representative beneficial microbial taxa at genus level. Data are expressed as mean ± SEM, different color graphics in each group represent duplicates. #: *p* < 0.05 compared to the control group; *: *p* < 0.05 compared to the MP group; *n* = 6–8.

Herein, we analyzed the effects of different heat-killed probiotic bacteria in pasteurized yogurt on gut microbiota composition in MP-sensitized mice (Figure 5). The diversity of gut microbiota was decreased (*p* < 0.05) in allergic mice of the MP group, as indicated by the Ace and Chao index, in contrast to control mice (Figure 5A). Even though the diversity was also lower in allergic mice receiving pasteurized plain and heat-killed BBMN68 pasteurized yogurt, the proportions of beneficial *Spirillum*, *Mycobacterium*, *Lactobacillus,* and *Prevotella* were higher than those in the MP group, as shown in Figure 5C. The PCoA plot based on the unweighted-unifrac distances of the OTUs was presented (Figure 5B). The MP group and the control group were separated, while the BBMN68 + MP group was distinctly separated from the MP group. In Figure 5C, the bacterial community structure showed that Bacteroidetes and Firmicutes were the major bacterial phyla, and Bacteroidetes were more dominant in the MP and all pasteurized yogurt treatment groups than the control group. At the genus level, the proportion of harmful bacteria, including *Clostridium* and *Cholephila* increased while beneficial *Spirillum*, *Mycobacterium*, and *Lactobacillus* dropped in the gut microbiota of mice in the MP group in contrast to the control group. The MP induced a gut environment favoring Th1/Th2 imbalance and inflammation by altering microbiota structure in mice; supplementation of pasteurized yogurt with and without heat-killed probiotics obviously mitigated this deleterious effect via increasing health-promoting bacteria proportion.

An LEfSE analysis was performed to compare the relative contributions of different taxa among different groups (Figure 5D). A total of 38 significant different taxa at the genus level among groups were determined. Compared with the control group, increased pro-inflammatory bacteria (*Tyzzerella*, *Clostridia,* and *Anaeroplasma*) were found in the intestinal tract of allergic mice in the MP group. The anti-inflammatory bacteria (e.g., *gRhodospirillales* and *Prevotellaceae*) were enriched in the Y + MP group. Beneficial bacteria *Lachnospiraceae* and *Eubacterium_siraeum_*group were detected in the LGG + MP group. Meanwhile, higher abundances of anti-inflammatory beneficial bacteria *Lactobacillus*, *Odoribacter*, *Eubacterium_brachy_*group, and *Parvibacter* were detected in the BBMN68 + MP group. A comparison of representative microbial taxa from these discriminative genera is shown in Figure 5E. Allergic mice receiving pasteurized yogurt containing heat-killed BBMN68 had significantly higher abundances (*p* < 0.05) of beneficial *Lactobacillus*, *CandidatusSaccharimonas*, *Odoribacter*, and *Parabacteroides* than those in the MP group.

**Figure 6:**
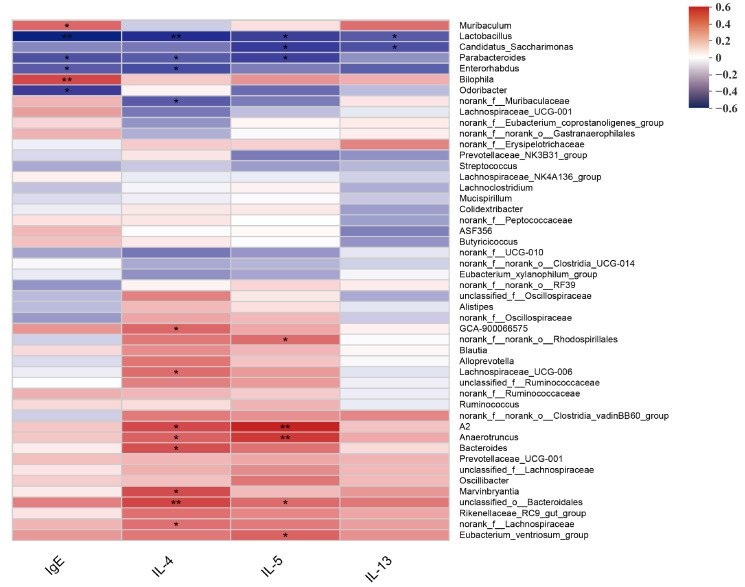
Pearson correlations between the relative abundance of gut microbiota and serum IgE, IL-4, IL-5, and IL-13 levels of allergic mice treated with pasteurized plain and heat-killed BBMN68 or LGG yogurt. * *p* < 0.05, ** *p* < 0.01.

The Pearson correlation between gut microbial composition and serum IgE and Th2 cytokines was analyzed (Figure 6). Significant negative correlations were detected between allergic responses and genus involved with immune function, such as *Lactobacillus* and serum IgE, IL-4, IL-5, and IL-13; *Candidatus_Saccharimonas* and serum IL-5, IL-13; *Parabacteroides* and IgE, IL-4, IL-5; *Enterorhabdus* and IgE, IL-4; *Odoribacter* and IgE; as well as *norank_f__Muribaculaceae* and IL-4. In particular, *Lactobacillus*, *Candidatus_Saccharimonas*, *Odoribacter*, and *Parabacteroides* were enriched in the gut of allergic mice after heat-killed BBMN68 pasteurized yogurt intake (Figure 5E).

## 4. Discussion

The rising prevalence of allergic airway disease has become a threat to public health that affects millions of people and causes high economic costs in many countries. Exposure to airborne allergen sources is inevitable and brings challenges to life quality improvement. In this study, we found the protective effects of heat-killed *B. longum* BBMN68 in pasteurized yogurt in a MP-induced allergic sensitization mouse model. The results demonstrated that oral supplementation of heat-killed BBMN68 decreased serum IgE levels, adjusted Th1/Th2 immune balance, alleviated airway inflammation, promoted airway remodeling, and reshaped the gut microbiota composition without undesirable side effects.

As a typical source of good bacteria and nutrients, yogurt possesses multiple health-promoting effects, including immunologic and metabolic benefits [22,23,24]. It has been shown that fresh and pasteurized yogurt had no significant differences in immunological parameters [25]. Pasteurized yogurt has many advantages; for example, it can be temporarily stored at room temperature, which greatly expands its potential use. Moreover, yogurts supplemented with additional probiotics and nutrients such as vitamin D display combined effects to enhance positive outcomes via modulating gut microbiota [26]. In this study, oral supplementation of pasteurized yogurt with heat-killed BBMN68 exhibited improved protection against MP-induced allergies compared with pasteurized plain yogurt, which indicated that yogurt can be a nutritious delivery medium for postbiotics.

Heat-killed probiotics, also known as postbiotics, have been used as a novel strategy for the prevention and treatment of many immune and gastrointestinal disorders, such as allergic airway disease, food allergies, and necrotizing enterocolitis. Oral administration of heat-inactivated *Lactobacillus plantarum* K37 showed anti-allergic effects by modulating airway hyperresponsiveness in ovalbumin-sensitized BALB/c mice [27]. Heat-killed *E. faecalis* YM-73 and *L. salivarius* AP-32 switched the immune response from a Th2 toward a Th1 response via increasing Th1-associated cytokines and reducing Th2-associated cytokines in Caco-2 cells [28]. Our results showed that heat-killed LGG significantly downregulated the Th2 cytokines but not the total IgE level, which is consistent with a previous study [11]. Numerous studies suggest that the major health-promoting roles of beneficial microbes, for example LGG, are linked with their non-viable byproducts, including cell-wall composition (mainly peptidoglycan and lipoteichoic acids), exopolysaccharides, surface-layer proteins, cell-free supernatants, bacteriocins, and metabolites such as short-chain fatty acids (SCFAs). These by products have the potential to facilitate the growth of beneficial commensal bacteria, hinder the growth and adherence of pathogenic bacteria, and induce anti-inflammatory and/or antioxidant activity [10,29,30,31,32,33]. Even though the functional constituents and their biological activities of *B. longum* BBMN68 as a postbiotic have yet to be fully elucidated, a previous study indicated that its cell wall fraction is the major effective component compared to the cell-free extract in a model of LPS-stimulated macrophage-like cells [19]. In this study, the protective effects of heat-killed LGG were less pronounced than those of heat-killed BBMN68, which may be due to their strain-specific immunomodulation and conclusion that heat-killed BBMN68 has a better capacity for reducing sensitization severity. The temperature and duration of heat treatment have a critical impact on the immune-related properties of probiotics cell surfaces. Li et al. found that heat treatment of LGG at 80 °C for 20 min decreased LPS-induced proinflammatory mediators and increased anti-inflammatory mediators in rats [14]. Another study on *Bifidobacterium longum* indicated that heat treatment of CECT-7347 at 121 °C for 20 min exhibited antioxidant, anti-inflammatory, and gut-barrier protection properties [34]. Scanning electron microscopy analysis of the LGG cell wall after spray-drying revealed that the pili were removed after heating, which resulted in reduced adherence capacity [35]. Further research is needed to fully understand the impact of heat treatment on the immunomodulatory properties of LGG and BBMN68 and determine the optimal conditions for preserving probiotic properties.

Eosinophilic airway inflammation is predominant in both allergic and non-allergic sensitization [36]. The oral administration of heat-killed BBMN68 in pasteurized yogurt significantly reduced BALF eosinophils, largely due to the decreased Th2-associated cytokines IL-4, IL-5, and IL-13 levels in serum, and therefore may modulate the contraction and length adaptation of airway smooth muscle cells, contributing to the improvement of diminished airflow obstruction. Meanwhile, Th1 and Th17-related immune responses were not observed in this research, which implicates that BBMN68 maintains systemic immune balance primarily through downregulating the Th2 response. The suppression of IL-4 and IL-13 also accounts for lower IgE production [1]. The serum IgG production was not altered with postbiotic treatments; similar results have been reported on probiotics using different allergic models [4,11]. It was speculated that successful treatment of allergic disease does not necessarily depend on decreased allergen-specific IgG1 or IgE antibody levels [4,37]. In addition, systemic total IgG is not representative of IgG subclass profiles; the MP allergenic protein Art v 1-specific IgG should be determined in future studies. In recent years, the role of eosinophils in the pathogenesis of severe allergic inflammation has been recognized, which makes anti-eosinophil therapy a promising target [38]. Our result could lead to the development of new therapies using postbiotics to target eosinophilic airway inflammation. In addition, the correlations between immune reactions, inflammation, and specific strains of gut bacteria were explored. The links were found between specific genus and serum cytokine levels, which provide more evidence for effective anti-IL-5 or anti-IL-4 approaches by precisely targeting gut microbiota for allergic airway disease prevention and treatment.

The phenomenon in this study also supported the idea of a “diet-gut-lung axis”. Emerging evidence has revealed the communication between allergic inflammation and microbiota in the gut and lung after probiotic and prebiotic administration via oral or intranasal routes, thus leading to a reduction in severity and susceptibility in animals and humans [11,39,40]. The microbiota, both in the gut and lung, plays important roles in the development and regulation of allergic inflammation by influencing the maturation and function of the systematic immune system [41,42]. The constituents and metabolites derived from the gut microbiota can be the key signaling molecules of the immune system. Among them, SCFAs (e.g., acetate, butyrate, and propionate) are recognized as the most important mediators [43]. They can bind to G-protein-coupled receptors (GPCR) such as 41 and 43 located on the surface of intestinal epithelial cells and therefore activate regulatory T cells (Treg) and inhibit NF-kB activity, leading to anti-inflammatory effects and gut homeostasis maintenance [44]. For example, butyrate is reported to induce IL-10 and IL-18 production through GPR109A to inhibit colonic inflammation and inflammation-associated cancers [45]. Moreover, the SCFAs produced in the gut can be transported to the blood stream and influence lung and bone marrow homeostasis. SCFAs in the lung inhibit the activity of histone deacetylase and promote the mTOR-S6K pathway to regulate T cell differentiation and cytokine production to combat allergic airway diseases [46]. SCFAs in the bone marrow, a major site of innate and adaptive immune cell development, can promote the differentiation of hematopoietic stem cells and the production of monocyte and DC progenitors (MDPs) and common DC precursors (CDPs), which can migrate to the lung to mitigate Th2 cell-mediated inflammation [47,48]. Herein, a negative correlation was found between the genus involved with SCFA-producing bacteria *Lactobacillus*, *Parabacteroides*, and *Odoribacter* and serum antibodies and Th2 cytokines, indicating that heat-killed BBMN68 may influence the systemic immune system by favoring SCFA-producing bacteria growth. Previous studies have shown that dysbiosis of the gut microbiome stimulates local and systematic inflammatory pathways and leads to airway hyperresponsiveness [42]. In this study, even though lower bacterial community diversity was observed, the yogurt containing heat-killed BBMN68 modified the gut microbiota composition by promoting the abundance of beneficial bacteria such as *Spirillum*, *Mycobacterium*, *Lactobacillus*, and *Prevotella*. Overall, the improved inflammatory conditions in the lung may result from the gut homeostasis maintained by inactivated *B. longum* BBMN68 intake. The influence of heat-killed BBMN68 on the lung microbiome should be assessed in future studies.

This research provides evidence that pasteurized yogurt containing BBMN68 exhibits immunomodulatory activity in a mouse model and highlights its potential application in the protection of pollen-induced allergic airway inflammation. However, the applicability of such findings to humans is uncertain and requires further research. More clinically relevant parameters of allergic inflammation-associated diseases typically observed in humans should be determined to improve the translational value of data from animal models. Based on that, clinical trials of different human life stages, especially early life, deserve further exploration to determine the effects of pasteurized yogurt containing BBMN68 on allergic airway diseases.

In conclusion, this study testified that treatment of allergic sensitization by oral administration of yogurt containing heat-killed BBMN68 is valid in an MP-induced allergic mouse model and that heat-killed BBMN68 can be used as a postbiotic. The optimal dose and most effective mode of administration via nasal or oral routes still require further investigation. More importantly, future studies on the functional constituents of BBMN68 and the mechanisms of their biological activities are essential for its potential application.

## Data Availability

All related data and methods are presented in this paper. Additional inquiries should be addressed to the corresponding author.
